# Knowledge graph and frontier trends in melanoma-associated ncRNAs: a bibliometric analysis from 2006 to 2023

**DOI:** 10.3389/fonc.2024.1439324

**Published:** 2024-11-26

**Authors:** Ru Wang, Xiao-yan Zhu, Yi Wang

**Affiliations:** ^1^ Department of Pediatrics, Xinzhou District People’s Hospital, Wuhan, Hubei, China; ^2^ Sanquan College of Xinxiang Medical University, Xinxiang, Henan, China; ^3^ The Fifth People’s Hospital of Hainan Province, Affiliated Dermatology Hospital of Hainan Medical University, Haikou, Hainan, China; ^4^ The Department of Dermatology, Xiangya Hospital, Central South University, Changsha, Hunan, China

**Keywords:** melanoma, non-coding RNA, miRNA, lncRNA, bibliometric analysis

## Abstract

**Objectives:**

Malignant melanoma (MM) is a highly malignant skin tumor. Although research on non-coding RNAs (ncRNAs) of MM has advanced swiftly in recent years, no specific bibliometric analyses have been conducted on this topic. The present study aims to summarize the knowledge graphs and frontier trends and to provide new perspectives and direction of collaboration for researchers.

**Method:**

Research data on melanoma and ncRNA published from January 1, 2006 to October 9, 2023 were retrieved and extracted from Web of Science. R Studio (Version 4.3.1), Scimago Graphica (Version 1.0.36), VOSviewer version (1.6.19), and Citespace (6.2.4R) were used to analyze the publications, countries, journals, institutions, authors, keywords, references, and other relevant data and to build collaboration network graphs and co-occurrence network graphs accordingly.

**Results:**

A total of 1,222 articles were retrieved, involving 4,894 authors, 385 journals, 43,220 references, 2413 keywords, and 1,651 institutions in 47 countries. The average annual growth rate in the number of articles was 25.02% from 2006 to 2023; among all the journals, Plos One had the highest number of publications and citations, which are 42 publications and 2,228 citations, respectively. Chinese researchers were the most prolific publishers in this field, having published a total of 657 articles, among which 42 were published by Shanghai Jiao Tong University, which was the most productive institution. In recent years, the most explored keywords included long non-coding RNAs, immunotherapy, and exosm. According to the timeline chart of reference co-citation, “functional role” has been the most explored hotspot since 2015, and human cancer is a newly emerged hotspot after 2021.

**Conclusion:**

Through a bibliometric analysis, this study included all publications on ncRNAs and melanoma that were published in English from 2006 to 2023 in Web of Science to analyze the trends in the number of publications, international research focuses, and the direction of collaboration. The results of this study may provide information on knowledge graph, frontier trends and identify research topics in melanoma. More current research proved that ncRNA plays a crucial role in the biological behavior of melanoma including proliferation, invasion, metastasis, drug resistance, etc. With the development of research on ncRNA and melanoma, ncRNA may great potential in development of early diagnosis, targeted therapy and efficacy evaluation in the future. The results of this study also provide new perspectives and research partners for researchers in this field.

## 1 Introduction

Melanoma is a highly aggressive malignant tumor of the skin with a high mortality rate; its incidence only accounts for 4% of all skin cancers, but it accounts for more than 75% of skin cancer-related deaths (1). Furthermore, an individual’s risk of developing melanoma has risen from 1/500 in 1935 to 1/50 in 2023 (2). It is estimated that there have been 325,000 new cases of melanoma and 57,000 deaths from melanoma in 2020 globally, and in most regions of the world, the incidence of melanoma is higher in males than in females (3). At present, the cause of melanoma is still unknown, and it has been found that environmental and genetic factors, as well as ultraviolet radiation, may be the main contributors to its development (4). As MM is prone to vascular invasion, it is highly likely to metastasize and relapse, leading to a poor prognosis (5). Since 2011, the U.S. Food and Drug Administration (FDA) has approved 10 new therapies for the treatment of metastatic melanoma, which has seen significant advances in the treatment of this disease due to the introduction of targeted immunotherapy and targeted therapy (6). Studies have shown that treatment with immune checkpoint inhibition therapy can improve the survival of patients with metastatic melanoma (7), but there are still problems such as drug resistance (8) and immune-related adverse events [e.g., rheumatic, neurological, gastrointestinal and skin diseases (9)]. Therefore, more research is still needed for the treatment and prognosis of melanoma.

ncRNAs are non-coding RNAs that generally do not encode proteins; classical ncRNAs include microRNA, lncRNA, and cirRNA (10). While ncRNAs affect translation and splicing, they also influence the modification of other RNA molecules. NcRNAs are widely recognized as universal regulatory factors for a variety of cancer hallmarks (e.g., proliferation, apoptosis, invasion, metastasis, and genomic instability) (11). Despite recent advances in the research of cancer treatment, resistance to chemotherapy, radiotherapy, targeted therapy and immunotherapy still remains a major setback. Recent studies have shown that ncRNAs play an important role in resistance to different cancer therapies by reconnecting important signaling pathways (11). It has also been found that they can affect the development and progression of melanoma through different mechanisms, which may have practical implications in the diagnosis and treatment (12).

Different from traditional research methods, Bibliometrics is a new methodology that employs mathematical and statistical techniques to provide researchers with the knowledge structure of the subject field by analyzing research literature in a specific field, including the measurement indicators of countries, journals, institutions and keywords, thereby suggesting potential directions and challenges in this field (13). Bibliometrics also has made great contributions to the formation of disease treatment and clinical guidelines (14, 15). At present, a large amount of research has been accumulating in MM and ncRNA. However, to the best of our knowledge, there have been few reports involving analyses of progress and frontier trends in the study of MM and ncRNA. Thus, using CiteSpace and VOSviewer software and based on research literature on melanoma and ncRNAs from Web of Science, we conducted a systematic investigation of scientific outputs related to ncRNAs and melanoma from 2006 to 2023, in order to shed light on current trends of research in this field. While exploring collaborations between authors, institutions, and countries, we also comprehensively analyzed the keywords and references to identify the research priorities and hotspots, with a view to providing new ideas for the clinical diagnosis and treatment of melanoma.

## 2 Methods

**Figure 1:**
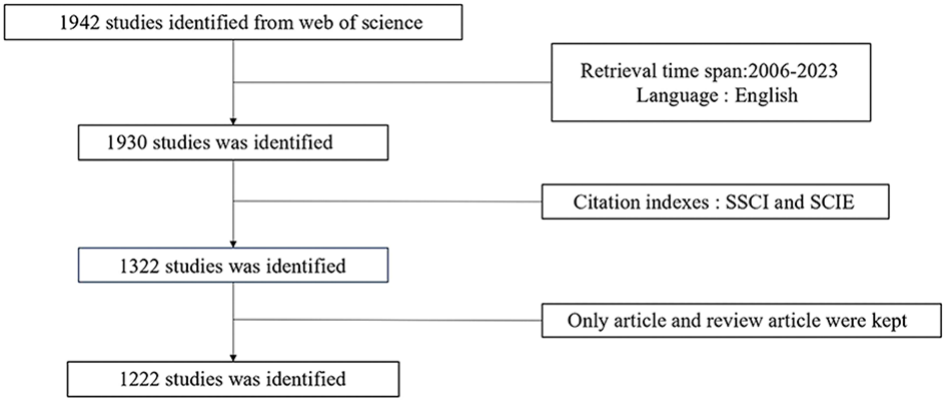
The flow diagram of literature search and selection process.

Literature related to ncRNA and melanoma published from 2006 to 2023 was retrieved from Web of Science, and publications meeting the following criteria were included in further analysis: 1) TS=(“melanoma”) AND TS=(“Non-coding RNA” OR “ncRNA” OR “Untranslated RNA” OR “Micro RNA” OR “miRNAs” OR “Circular RNA” OR “circRNA” OR “Piwi-interacting RNA” OR “Long non-coding RNA” OR “lncRNA” OR “lincRNA” OR “long intergenic non-protein coding RNA” OR “lncRNAs” OR “ceRNA” OR “competing endogenous RNA”); 2) the publications should be articles and review articles; 3) the articles were published from 2006 to 2023; 4) the articles were published in English. Exclusion criteria 1) not relevant to the research topic 2) duplicate articles and review articles. 2 researchers collated the included studies and extracted the data independently. The data were verified by 2 researchers to ensure the accuracy and completeness of the results. Any disagreements were resolved by discussion or third-party discussion. The research procedure is presented in **Figure 1**. R Studio (Version 4.3.1), ScimagoGraphica (Version 1.0.36), VOSviewer (Version 1.6.19), and CiteSpace (Version 6.2.4R) software were used for the visualized analysis of selected literature and the creation of collaboration network and co-occurrence network graphs of countries/regions, institutions, authors, keywords, and references as well as co-citation graph.

This study is based on published articles from January 01, 2006 to October 9, 2023, which are available in public databases and do not involve human or animal studies or experiments. Therefore, this study does not require ethical approval.

## 3 Results

**Figure 2:**
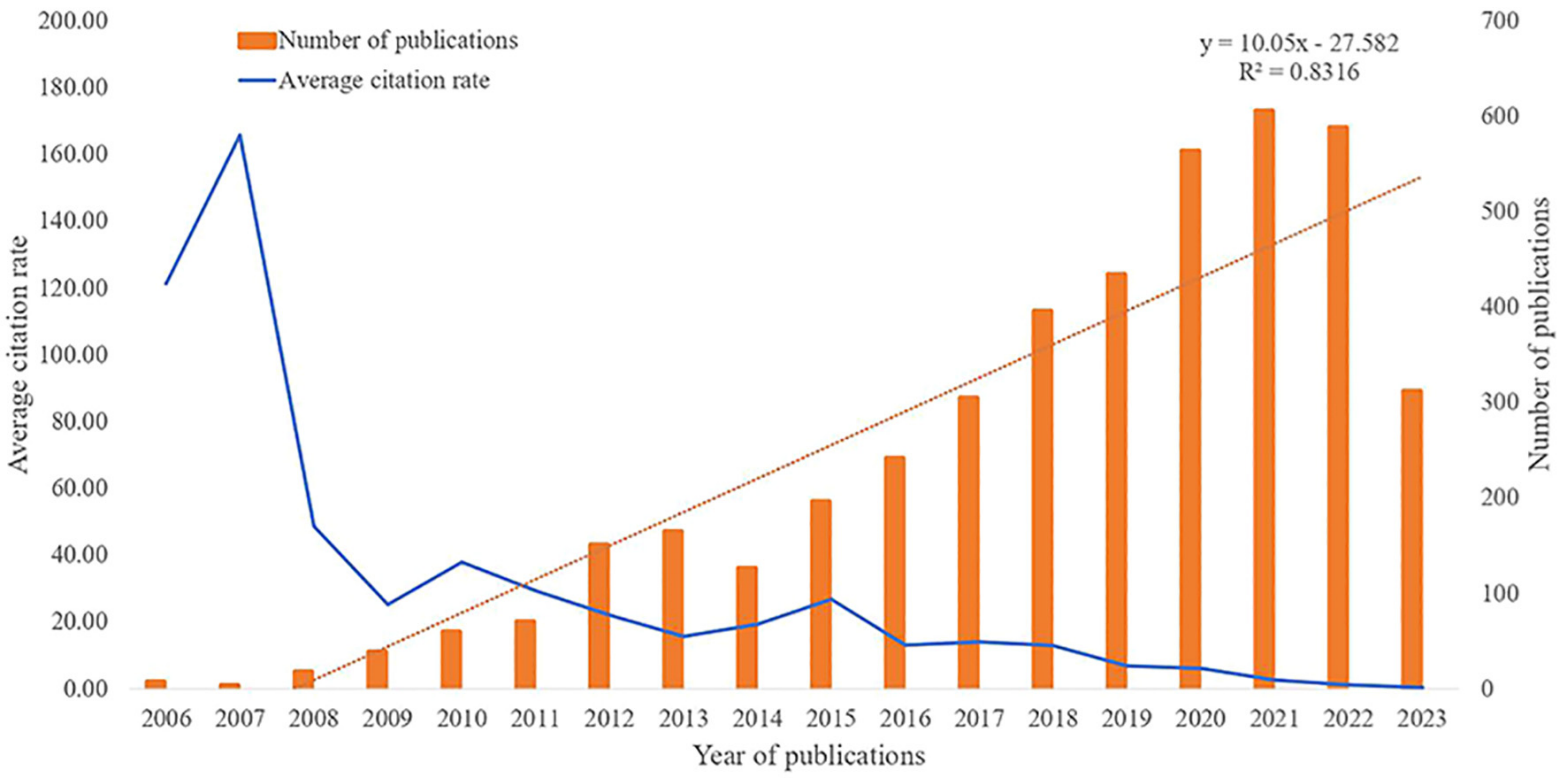
Publications related to melanoma-associated ncRNAs from 2006 to 2023 and the average number of citations.

A total of 1,222 articles related to melanoma and ncRNA published after 2006 were retrieved. Overall, the number of publications per year has been increasing year by year, with an average growth rate of 25.02%. The cumulative total number of citations of these articles was 42,264, with an average frequency of 34.58 citations per article. We also found a statistically significant association between the number of publications and time (R^2^ = 0.8316, **Figure 2**). By the time of this research, 89 articles have been published in 2023, and based on our linear prediction, 154 articles may be published by the end of 2023. Interestingly, only one article was published in 2007, but this article has attracted an average of 580 citations. 2018 was the first year that more than 100 articles were published, and the number of publications has remained above 100 in subsequent years. The increase in publications suggests that ncRNAs-associated MM has become a new topic of interest, and there are still many unknowns to be further investigated.

### 3.1 Countries/regions and co-authorship

**Figure 3:**
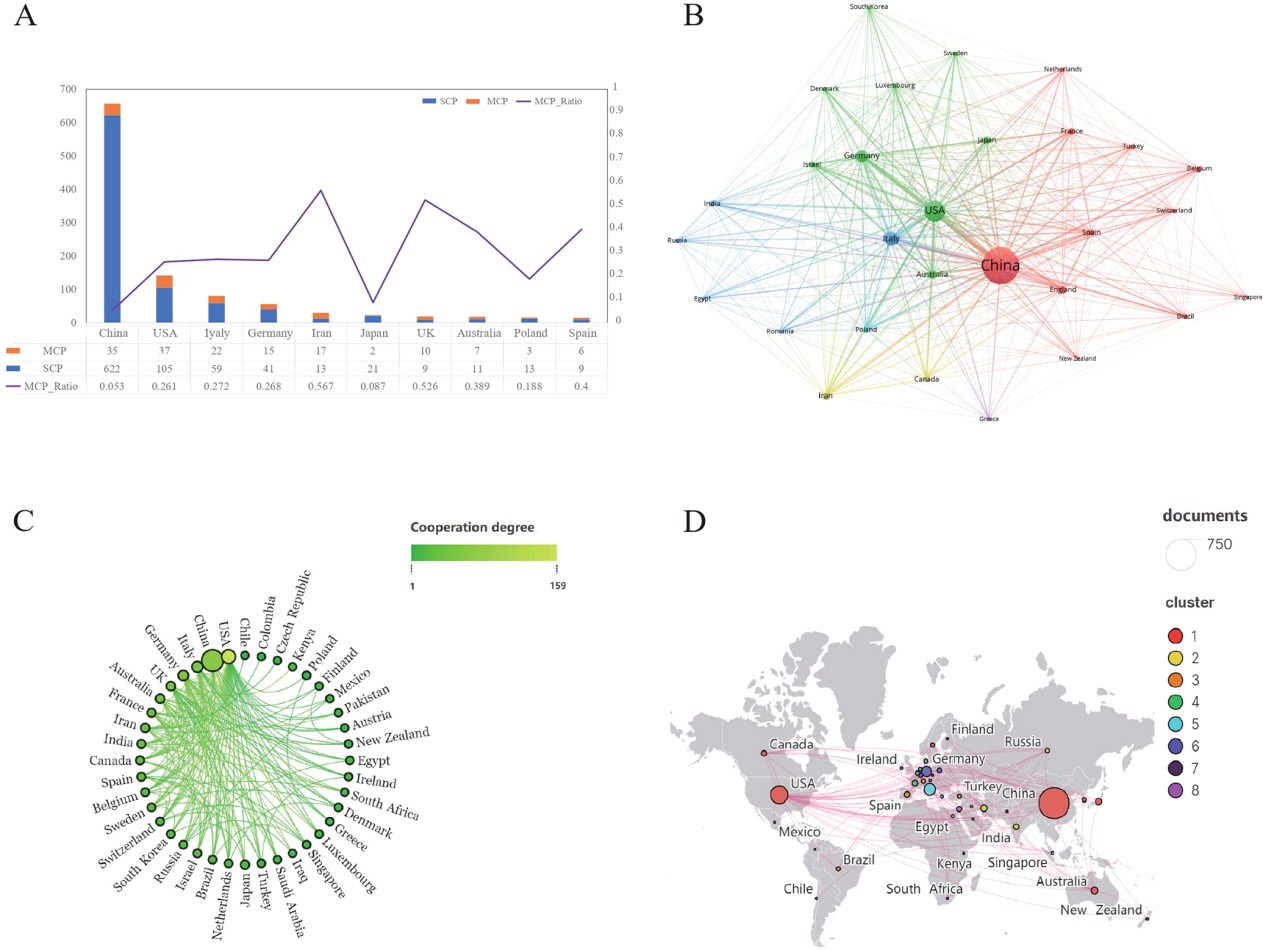
Analysis of the publications by countries. **(A)** The MCP and SCP graphs of the top 10 countries regarding the number of publications. **(B)** The visual network diagram created using VOSviewer. The nodes represent different countries, and the size of the nodes indicates the number of citations. The links between nodes represent the collaboration between countries, and the thickness of lines indicates citation strength. **(C)** The collaboration between countries. Denser lines indicate more countries in collaboration. **(D)** The countries in collaboration marked on the world map.

The 1,222 articles were from 47 countries, and the top 10 countries regarding the number of publications are presented in **Table 1**. China was the largest contributor, having published 672 articles, accounting for 55% of all the articles, with a total number of citations being 15,074. The US ranked second in the number of publications (217 articles), but their publications have the highest total number of citations, which was 15,695; this was followed by Italy (94 publications) and Germany (73 publications). According to the analysis of multiple country publications (MCP) ratio, Iran and the UK were more likely to collaborate with other countries (**Figure 3A**). A visualized analysis was conducted using the VOSviewer software on the countries with at least five publications on melanoma and ncRNAs, with countries showing the same color having closer collaboration (**Figure 3B**). Scimago Graphica was used to further demonstrate the collaboration between countries, with denser lines indicating closer collaborations; it indicates that the US has the largest number of research partners (**Figures 3C, D**).

**Table 1:** Top 10 journals containing the largest number of publications in the present study.

Rank	Country	Publications	Citations	Average Article Citations
1	CHINA	672	14670	22.30
2	USA	147	12525	88.20
3	ITALY	85	2316	41.40
4	GERMANY	60	2122	26.20
5	IRAN	31	1002	52.70
6	AUSTRALIA	23	925	40.20
7	JAPAN	19	865	48.10
8	UK	19	794	26.50
9	FRANCE	16	620	62.00
10	SPAIN	15	612	47.10

### 3.2 Institutions

**Figure 4:**
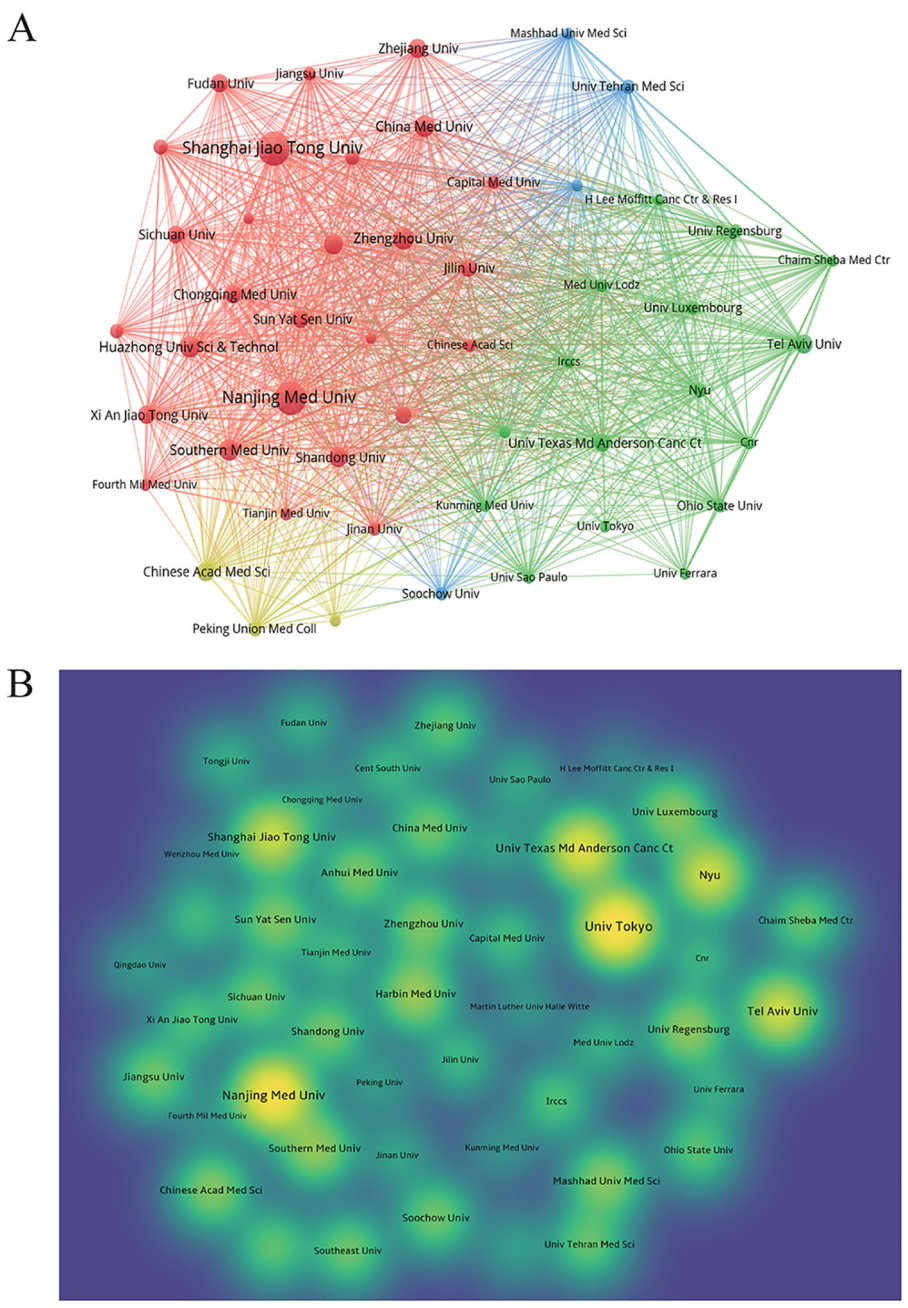
Analysis of publications by organizations. **(A)** The visual network diagram created using VOSviewer. The nodes represent different institutions, and the size of the nodes indicates the number of publications. The links between nodes represent the collaboration between institutions, and the thickness of lines indicates the strength of collaboration. **(B)** The density plot created using VOSviewer. The size of circles represents the number of publications, and the shade of colors represents the number of citations, with yellower circles indicating more citations.

The articles were published by a total of 1,651 institutions, and the top 10 institutions regarding the number of publications are presented in **Table 2**. The analysis of publications from 50 institutions showed that the top three institutions regarding the number of publications were Nanjing Medical University (106 articles), Shanghai Jiao Tong University (91 articles), and China Medical University (68 articles); publications of Nanjing Medical University achieved the largest number of citations (1,653) (**Figure 4A**). Interestingly, the University of Tokyo only had nine publications, which, however, were cited as many as 1,783 times. Institutions with at least eight publications were visually analyzed using the V OSviewer software; the nodes represent different institutions, with institutions showing the same color having closer collaborations (**Figure 4B**).

**Table 2:** Top 10 countries with the largest number of publications related to melanoma-associated ncRNAs.

Rank	Journal	Publication	Citations	Citations per publication	Impact factor (2022)	JCR
1	*Plos One*	42	2230	53.09	3.7	Q2
2	*International Journal of Molecular Sciences*	38	505	13.29	5.6	Q1
3	*Cancers*	37	497	13.43	5.2	Q2
4	*Oncotarget*	34	1791	52.68	5.2	Q1
5	*Frontiers in Oncology*	30	331	11.03	4.7	Q2
6	*Scientific Reports*	21	660	31.43	4.6	Q2
7	*Oncology Letters*	20	574	28.70	2.9	Q3
8	*European Review for Medical and Pharmacological Sciences*	19	228	12.00	3.3	Q3
9	*Frontiers in Cell and Developmental Biology*	15	224	14.93	5.5	Q1
10	*Aging-US*	14	173	12.36	5.2	Q2

### 3.3 Journals

**Figure 5:**
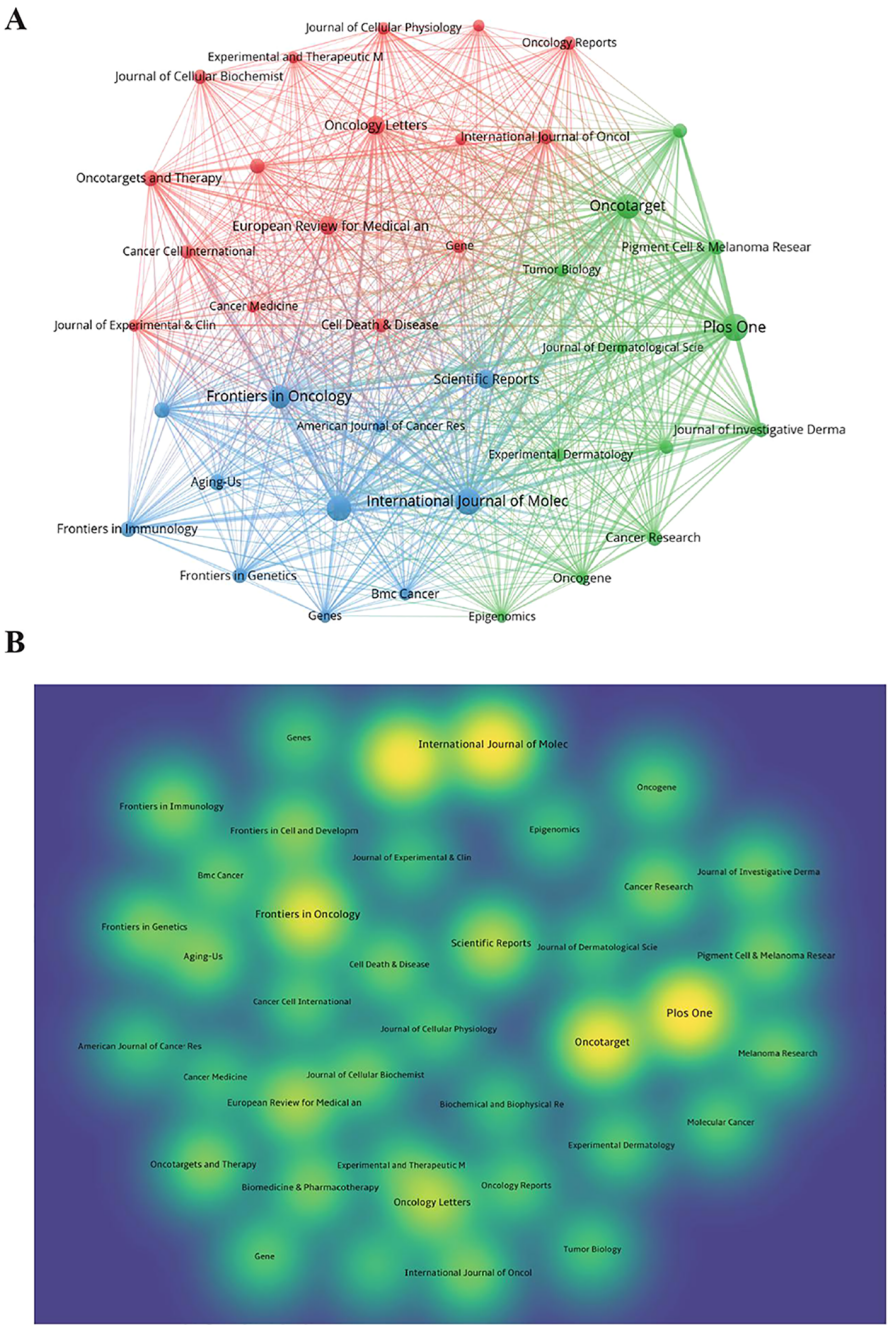
Analysis of publications by Journals and authors. **(A)** The visual network diagram created using VOSviewer. The nodes represent different journals, and the size of the nodes indicates the number of publications. The links between nodes represent collaborations, and the thickness of lines indicates the strength of collaboration. **(B)** The density plot created using VOSviewer. The circles represent different journals, and the size of circles indicates the number of publications; the shade of colors represents the number of citations, with yellower circles indicating more citations.

The 1,222 articles were published in 385 journals, and the top 10 journals regarding the number of publications are presented in **Table 3**. Plos One has the largest number of publications (42 articles), followed by the International Journal of Molecular Sciences (IJMS, 38 articles) and Cancers (37 articles). Impact factor (IF) is a key indicator for evaluating the influence of an academic journal. Among the top 10 journals, IJMS has the highest IF, indicating its high impact in the field of melanoma and ncRNAs. A total of 39 journals, each of which included at least eight relevant articles, were visually analyzed using the VOSviewer software (**Figure 5A**). The results showed that the journals with the largest number of citations were Plos One and Oncotarget, with 2,230 and 1.791 citations, respectively, suggesting that the above two journals were popular in the field of melanoma and ncRNAs (**Figure 5B**).

**Table 3:** Top 10 institution with the largest number of publications related to melanoma-associated ncRNAs.

Rank	Institution	Publications	Citations
1	ShangHai JiaoTong University	42	1016
2	NanJing Medical University	41	1653
3	ZhengZhou University	23	577
4	Huazhong University of Science and Technology	22	311
5	Southern Medical University	21	675
6	China Medical University	21	505
7	Central South University	20	285
8	ShangDong University	19	512
9	Tel Aviv University	18	1067
10	Chinese Academy of Medical Sciences	18	427

### 3.4 Authors

The top 10 core authors regarding the number of publications are presented in **Table 4**. Of the top 10 authors, six were from China; among them, Xian-qun Fan and He Zhang made the greatest contribution, having published 13 articles. They were followed by Stephanie Kreis and Shengfang Ge, who published 11 articles.

**Table 4:** Top 10 authors with the highest number of papers in the present study.

Rank	Title		DOI	Total citations	Year	Journal	Impact factor (2022)
1	Genome-scale transcriptional activation by an engineered CRISPR-Cas9 complex		10.1038/nature14136	1689	2015	*Nature*	64.8
2	MicroRNAs exhibit high frequency genomic alterations in human cancer		10.1073/pnas.0508889103	842	2006	*PNAS*	11.1
3	Non-coding RNAs as drug targets		10.1038/nrd.2016.117	666	2017	*Nature Portfolio*	120.1
4	Characterization of microRNA expression levels and their biological correlates in human cancer cell lines		10.1158/0008-5472.CAN-06-2698	580	2017	*Cancer Research*	11.2
5	*In Vivo* Identification of Tumor-Suppressive PTEN ceRNAs in an Oncogenic BRAF-Induced Mouse Model of Melanoma		10.1016/j.cell.2011.09.032	529	2011	*Cell*	64.5
6	Extracellular Vesicle Heterogeneity: Subpopulations, Isolation Techniques, and Diverse Functions in Cancer Progression		10.3389/fimmu.2018.00738	498	2018	*Frontiers in Immunology*	7.3
7	Chromatin structure analyses identify miRNA promoters		10.1101/gad.1706508	466	2018	*Genes & Development*	10.5
8	The Melanoma-Upregulated Long Noncoding RNA SPRY4-IT1 Modulates Apoptosis and Invasion		10.1158/0008-5472.CAN-10-4460	400	2011	*Cancer Research*	11.2
9	Melanoma addiction to the long non-coding RNA SAMMSON		10.1038/nature17161	392	2016	*Nature*	64.8
10	ANRIL, a long, noncoding RNA, is an unexpected major hotspot in GWAS		10.1096/fj.10-172452	353	2011	*Faseb Journal*	4.8

### 3.5 Keywords

**Figure 6:**
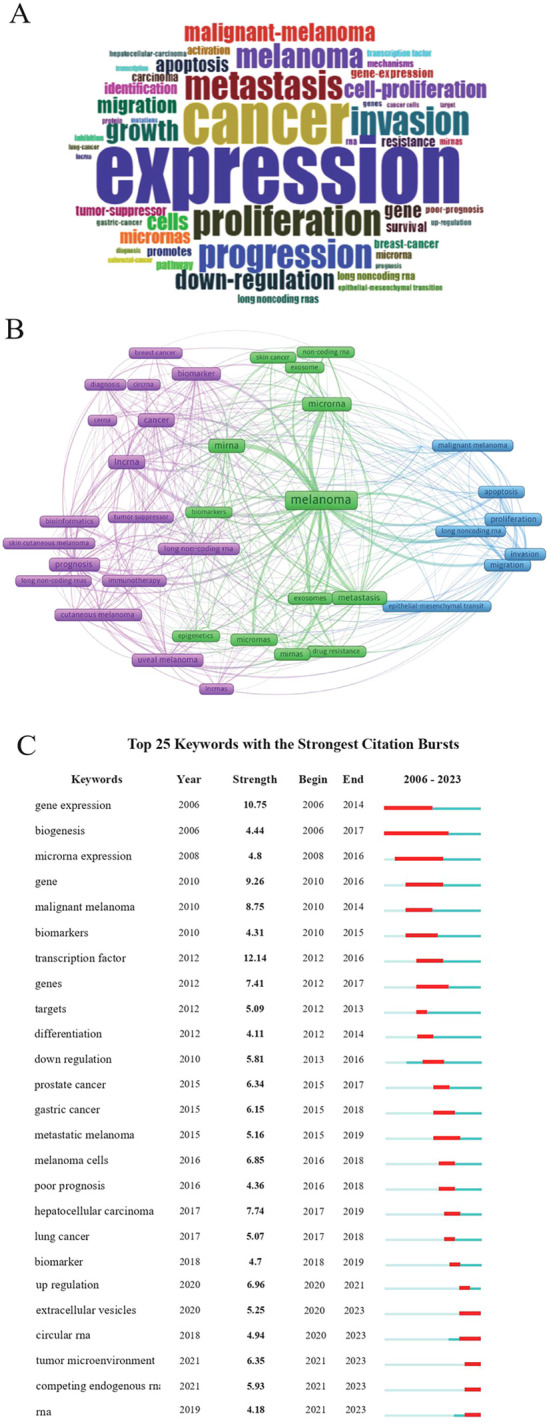
Analysis of keywords and research trends. **(A)** Presentation of keywords. Larger words indicated higher frequency of occurrence. **(B)** The visual graph created using VOSviewer. Each circle represents a keyword, with the size of circles indicating the frequency of occurrence. **(C)** Burst analysis of the top 25 keywords. The green line represents the timeline (from 2006 to 2023), and the red line represents the burst duration of keywords.

The 2,413 keywords in the 1,222 publications were analyzed using R Studio. The word cloud consisting of terms that appeared most frequently is presented in **Figure 6A**. Among all the keywords, expression, proliferation, and metastasis appeared most frequently. A total of 42 keywords, which appeared at least 12 times, were included in the network visualization (**Figure 6B**). Different from the results generated using R Studio, melanoma, microRNA, and lncRNA appeared more frequently. The top 25 keywords in terms of citation rate are presented in **Figure 6C**. The most frequently used keywords in the last three years included long non-coding RNAs, RNA, and immunotherapy, which was suggestive of new research hotspots.

### References

3.6

**Figure 7:**
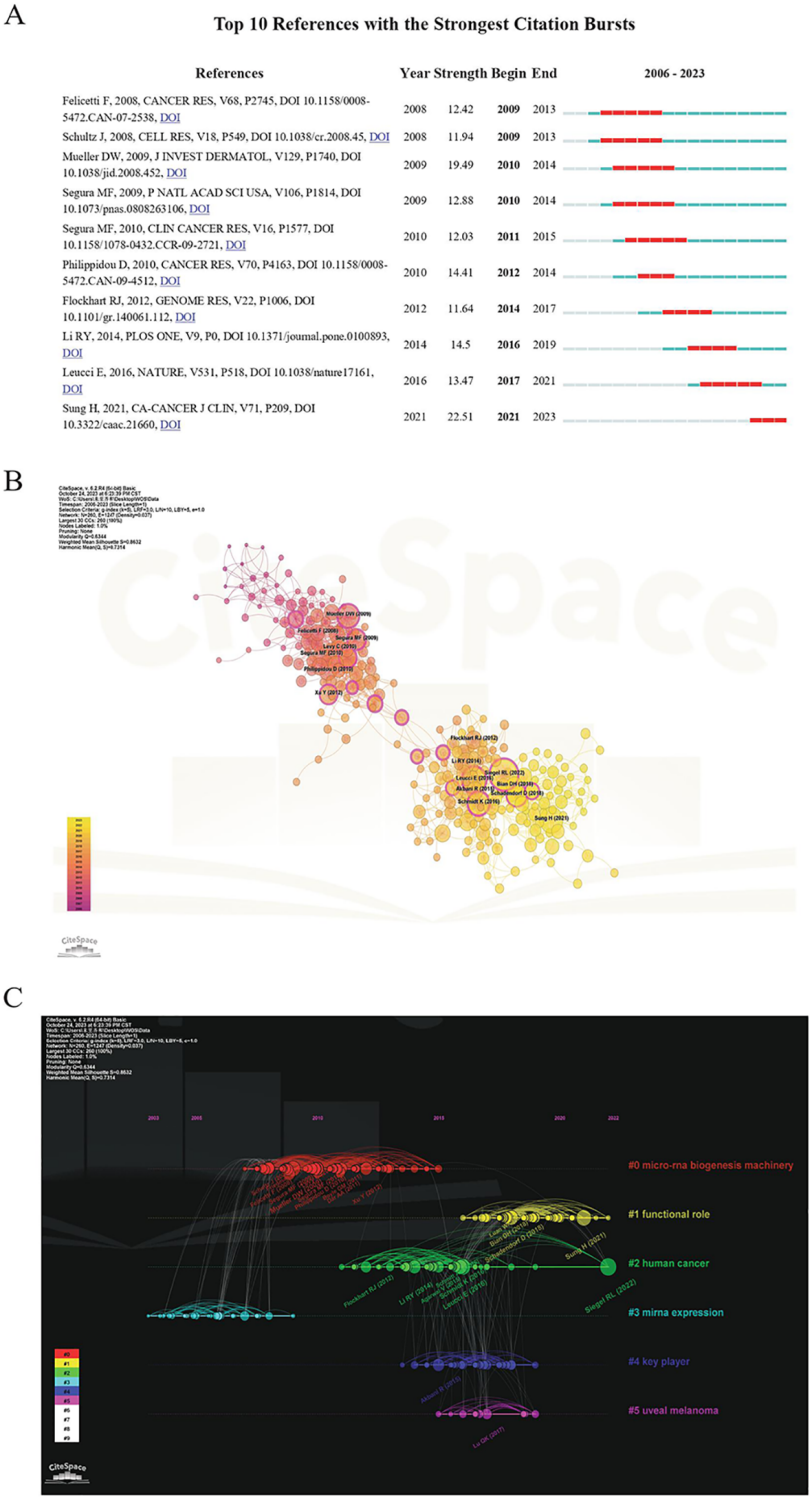
Co-cited reference analysis. **(A)** The top 10 references regarding citation burst based on CiteSpace analysis. **(B)** The co-occurrence structure of references created using CiteSpace. **(C)** The timeline of co-cited references related to the subject matter.

A total of 43,220 references were cited in the 1,222 publications. The highly cited articles are often important references and can sometimes be key articles that can set the foundation for a certain field. The top 10 references regarding the number of citations are presented in **Table 5**. Among them, an article titled *Genome-scale Transcriptional Activation by An Engineered CRISPR-Cas9 Complex*, which was published in Nature in 2014, was mostly cited (1,689 times). Burst analysis and cluster analysis on co-citations were conducted using the CiteSpace software. The top 10 co-cited references in terms of citation rate from 2006 to 2023 are presented in **Figure 7A**. The strength of the bursts ranged from 11.64 to 22.51, and the most frequently cited article was *Global Cancer Statistics 2020: GLOBOCAN Estimates of Incidence and Mortality Worldwide for 36 Cancers in 185 Countries* (strength: 22.51; publication year: 2021). The co-occurrence of references is shown in **Figure 7B**. The co-cited references were clustered into six directions to facilitate the observation of hotspot evolution and the exploration of the evolutionary track of this field (**Figure 7C**). A cluster was deemed reliable if the Q value was above 0.3 and the S value was above 0.7. “Functional role” has been a research hotspot since 2015 and peaked in the recent couple of years for studies of human cancers.

**Table 5:** Top 10 co-cited references related to melanoma-associated ncRNAs.

Rank	Author	Institution	Documents	Citations
1	Xian-qun Fan	Shanghai Jiao Tong University	13	417
2	He Zhang	Tongji University	13	385
3	Kreis stephanie	University of Luxembourg	11	622
4	Sheng-fang Ge	Shanghai Jiao Tong University	11	380
5	Wen-kang Luan	Nanjing Medical University	10	506
6	Perera Ranjan j.	Johns Hopkins University	9	851
7	Hernando eva	New York University	9	744
8	Philippidou Demetra	University of Luxembourg	9	604
9	Jin-long Wang	Nanchang University	8	447
10	Ren-bing Jia	Shanghai Jiao Tong University	8	280

## 4 Discussion

The present study focuses on the field of ncRNAs and melanoma. In this study, we conducted a bibliometric analysis on relevant articles published from 2006 to 2023 in Web of Science, with the relationship between the number of publications and year of publication, country/region, institution, journal, author, keyword, and co-occurrence and co-citation of references being main indicators. Compared with systematic review and meta-analysis, this study is based on large datasets and rigorous analyses to explore the development trends and current hotspots, which may provide new perspectives and ideas for future studies.

For more than a decade, enthusiasm for research on ncRNAs and melanoma has continued to rise, with an overall upward trend in the number of publications; most of the articles were published after 2012, accounting for about 95.4% of all the publications. This field has attracted 4,894 authors from 47 countries/regions, who had a total of 1,222 articles published. Among all these countries, China and the US are lead contributors with regard to the number of publications and citations, reflecting the core status of the two countries. All the top 10 institutions regarding the number of publications are based in China, with Nanjing Medical University ranking first. However, instead of pursuing more publications, the UK and Iran tended to seek international collaborations; although they did not have as many publications, they are keen for external collaborations, which should also be taken into consideration. Studies on ncRNAs and melanoma have involved a variety of aspects and have been published in journals of different disciplines. Among them, the top 10 journals include four oncological journals, two multidisciplinary scientific journals, two cytological journals, one molecular biological and biochemical journal, and one pharmacological journal. With regard to authors, the article of Mirzaei and Hamed has the highest citation rate, and Fan Xian-qun and Zhang He have the largest number of publications, suggesting the great contributions made by these authors.

Co-citation analysis can effectively reveal the knowledge base and background of ncRNAs and MM. The top 10 co-cited references cited epigenetic, epidemiological, and molecular biological studies. Co-occurrence analysis on keywords helps to categorize key knowledge structures and hotspots. In this study, co-occurrence cluster analysis was performed on the top 100 keywords that appeared at least 20 times, which generated the following three main clusters.

### 4.1 ncRNA and biomarkers of melanoma

Co-citation analysis can effectively reveal the knowledge base and background of Cluster 1 consists of 31 high-frequency keywords, including “melanoma”, “exosomes”, “miRNA”, and “biomarkers”, suggesting that this cluster is focused on studies of biomarkers of melanoma. Specific ncRNAs can help to distinguish between patients with melanoma and healthy individuals or between patients with metastatic and non-metastatic melanoma. Early diagnosis of MM is critical to treatment outcomes, but effective markers are still lacking. As potential biomarkers for early diagnosis of melanoma, targeted therapy for tumors, and assessment of treatment efficacy, ncRNAs have received increasing attention.

MicroRNAs are small, non-coding RNAs 19-25 nucleotides in length, and they are involved in the regulation of a variety of biological processes. MicroRNAs are affected by aberrant epigenetic alterations in tumors, which can lead to their dysregulation and cancer formation. Recently, dysregulation of several microRNAs has been reported in different types of cancers. Circulating microRNAs can be stably present in the circulatory system and can be used as potential biomarkers for early diagnosis, treatment, and prognosis. A meta-analysis involving 648 patients with melanoma and 578 control subjects (16) found that circulating miRNA has high diagnostic power for melanoma, with sensitivity, specificity and AUC of 87%, 81% and 0.90, respectively, suggesting that circulating microRNA might be a non-invasive biomarker for the diagnosis of melanoma. Another retrospective analysis suggested that the circulating oncogenes miR-579-3p and oncomiR miR-4488 could be used to predict whether targeted therapies are appropriate for patients with BRAF-mutant melanoma (17). An earlier microarray miRNA analysis on melanocytes and melanoma cell lines derived from primary tumors or metastatic melanoma found that the expression of seven miRNAs (i.e., miR-133a, miR-199b, miR-453, miR-520f, miR-521, miR-551b, and miR-190) was downregulated, and experiments showed that the expression of miR-17-5p, miR-222, miR-181a, miR-194, miR-22 and miR-373 was downregulated in both melanocytes and melanoma cell lines (18). In addition, miR-185 and miR-1246 were shown to have high specificity and sensitivity in the diagnosis of metastatic cutaneous melanoma (19). It has also been found that miR-146a-5p is highly expressed in human melanoma brain metastasis, suggesting its metastasis-promoting effect (20). miRNAs are present in the blood at low concentrations, but their expression can be easily detected using standard quantitative real-time reverse transcription polymerase chain reaction (qRT-PCR) (21). Therefore, miRNAs can be used as potential biomarkers for melanoma.

As ncRNAs play a key role in most of the regulatory mechanisms of biological homeostasis and disease development by controlling gene expression at the transcriptional, post-transcriptional, and epigenetic levels, they have received great attention in preclinical and clinical studies in recent years. For instance, lncRNA PVT1 was found to be differentially expressed in cutaneous melanoma, which revealed that lncRNA PVT1 might promote the tumorigenesis and metastasis of melanoma by binding to EZH2 and regulating miR-200c expression (22). Thus, lncRNA PVT1 might be a potential target for the treatment of melanoma. Furthermore, Du et al. found that the expression of lncRNA LINC02249 was significantly increased in cutaneous melanoma specimens, and this high expression was associated with shorter overall and disease-specific survival in patients with SKCM, indicating that lncRNA LINC02249 might be a novel biomarker for predicting the prognosis and immune infiltration of SKCM (23). Studies also found that among ncRNAs, SAMMSON, TYRP1, SPRY4-IT1, UCA1, MALAT-1, HOTAIR, SLNCR1, ANRIL, and BANCR were also upregulated to some extent in melanoma specimens (24); considering the important role of ncRNAs in resistance and metastasis of cancer as well as autoimmune diseases, we also highlighted their role as promising prognostic and therapeutic targets for skin diseases. lncRNAs also have great potential for assessing the response to ICI and predicting clinical outcomes; for instance, some IC-lncRNAs, such as LINC00324, RP11-445H22, and RP11-25K19, can target proteins that mediate the interactions between T cells and tumor cells (25), indicating that lncRNA may also be a target for immunotherapy of cancer. Such studies have described the potential role of lncRNAs as markers of melanoma and possible targets for therapies and interventions.

Circular RNA (circRNAs) are a class of single-stranded non-coding RNAs characterized by a covalently closed loop structure; by binding to specific miRNAs or proteins, they are involved in a variety of biological and cellular functions. Recent studies have found that circRNAs are involved in various anti-tumor immune responses and regulation of immune cells (26), and they have also become biomarkers for predicting the prognosis of patients with cancer (27). Circ_0020710 is usually highly expressed in melanoma tissues, and high levels of circ_0020710 are found positively correlated with the malignant phenotypes and poor prognosis of patients with melanoma (28). In addition, it has been demonstrated that most circRNAs found in melanoma, such as circ_0020710 (28), circ_0025039 (29), circ_0002770 (30), and circ_0001591 (31), have oncogenic activity. In summary, ncRNAs have great potential to become early diagnostic and prognostic markers for melanoma, although they have not come into use at present.

### 4.2 Impact of ncRNAs on the biological behavior of melanoma

Cluster 2 consists of 30 high-frequency keywords, including “proliferation”, “migration”, “metastasis”, and “invasion”, indicating that this cluster mainly focuses on the impact of ncRNAs on the biological behavior of melanoma.

MicroRNAs are involved in almost all aspects of melanoma, including its formation, growth, angiogenesis, and metastasis, and play a crucial role in the development of many diseases, including cancer (32). MicroRNA-3662 (miR-3662) downregulates its target mRNA and is related to the inhibition of melanoma cell proliferation (33). It was found that the level of miR-221 was elevated in the serum of patients with metastatic melanoma, and this might be correlated with tumor thickness, which reflects the progression status of melanoma (34). Furthermore, high levels of miR-206 were detected in the serum of patients with advanced melanoma, which was found associated with aggressive disease progression and poor prognosis (35). MiR-143 is involved in metastatic and apoptotic pathways, suggesting that it may act as a tumor suppressor microRNA in melanoma (36). MiRNAs can play important roles as tumor suppressors and oncogenes in melanoma (37), including miR-137, miR-101, miR-26a, miR-196a, miR-125, the miR-200 family, miR-205, miR-203 and miR-211 (32). In addition, miRNA is also found to be related to drug resistance; for example, increased expression of miR-9-5p, miR-4443, and miR-4488 and decreased expression of miR-199b-5p and miR-204-5p were found in the development of drug resistance (17). All the above findings have highlighted that circulating miRNAs may be a suitable tool for predicting response to melanoma treatment and may be further developed as a clinical companion diagnostic.

LncRNAs can influence the biological behavior of MM through multiple mechanisms. For example, lncRNA SPRIGHTLY can regulate melanocyte function by promoting melanocyte proliferation and reducing melanocytogenesis via UCA1 (38); lnc-PKNOX1-1 can inhibit melanoma development by regulating IL-8, thereby significantly inhibiting the proliferation, migration, and invasion of melanoma cells (39), which suggested its potential as an early diagnostic and therapeutic target for melanoma; lncRNA ZEB1-AS1 plays its role by activating the expression of ZEB1 gene, thereby affecting the aggressiveness and phenotypic transformation of melanoma (40). It has been reported that high levels of HOTAIR are associated with poor prognosis in patients with melanoma, and HOTAIR can promote the growth and metastasis of melanoma cells by competitively binding to miR-152-3p, leading to the functional release of c-MET mRNA as well as the activation of the downstream PI3k/Akt/mTOR signaling pathway (41). However, another study found that H19 was highly expressed in melanoma tissues compared to normal paraneoplastic skin tissues, and the level of expression in tissues was significantly higher in patients with metastatic melanoma than in those without distant metastases, suggesting that H19 might regulate the growth, invasion, migration, and epithelial mesenchymal transition (MET) of melanoma cells (42). Xu et al. found that HOXA11-AS could regulate the proliferation, apoptosis, metastasis and EMT of melanoma cells by modulating the miR-152-3p/ITGA9 axis (43). HOXA11-AS can promote melanoma development and may serve as a promising biomarker for the diagnosis and treatment of cutaneous melanoma. In addition, SAMMSON can modulate epigenetic processes by regulating histones or DNA methylation, which is significantly upregulated in cancer; it also induces melanoma cells to develop drug resistance to BRAF inhibition by increasing oxidative phosphorylation (44).

CircRNAs are also involved in the biological behavior of MM. It has been found that an increase in circ_0020710 can promote the proliferation, migration, and invasion of melanoma cells *in vitro* and tumor growth *in vivo* (28). The absence of circRNA and CDR1as can drive melanoma cell invasion and metastasis by binding and sequestering the protein IGF2BP3 (45). Taken together, the impacts of ncRNAs on the biological behavior of melanoma are mainly focused on proliferation, invasion, metastasis, and apoptosis, while their effects on other aspects are rare and need to be further explored.

### 4.3 Epidemiological analysis of ncRNA and melanoma

Cluster 3 consists of 14 high-frequency keywords, including “risk”, “expression, and “immunotherapy”, suggesting that this cluster is mainly focused on the epidemiological influence on melanoma. A study in 2017 demonstrated that BASP1 might be a susceptibility locus for malignant melanoma due to its decreased expression in melanoma; however, various ncRNA mutations may also lead to susceptibility to melanoma development (46). According to the burst analysis of keywords, the aspects of immunotherapy, upregulation, tumor microenvironment, and competing endogenous RNA were most frequently explored in the last three years, with a focus on the impact of ncRNAs on melanoma. In the present study, through the analysis of 10 publications with the largest number of citations, we found that eight of them were related to genetics, suggesting that more attention has been given to genetic studies on melanoma. The BRAF mutant is the most common mutant type of melanoma, accounting for over 60% of all cases, and NRAS and NF1 have also been found as common mutation sites in melanoma (2). The development of immunotherapies for melanoma has achieved great success, however, there are still a variety of immune-related adverse events, such as low relative lymphocyte count, C-reactive protein elevation, diarrhea, and increased drug resistance (47, 48) The management of immune-related adverse events is still under investigation, but ncRNAs have the potential to serve as therapeutic targets for preventing melanoma recurrence or increasing the sensitivity of melanoma to current therapies.

## 5 Limitations

In summary, the field of ncRNAs and MM has been brought to the attention of an increasing number of researchers, and more rigorous epidemiological studies and explorations of related mechanisms are warranted to facilitate future development. However, there are still some limitations to this study. Firstly, this study only included articles published in English from the Web of Science, which might have introduced source bias or omission of important studies; thus, articles from other databases such as PubMed and Scopus will also be analyzed in future studies. Furthermore, our analysis was performed on the basis that all the publications had the same validity, and the lack of quality assessment for the included articles might have affected our results. Lastly, recently published high-quality articles have not received great attention due to low citation rates, which might have affected the accuracy of our findings.

## 6 Conclusion

In this study, we included all the articles related to ncRNAs and melanoma published in English from 2006 to 2023 obtained from Web of Science and conducted a bibliometric analysis on the trends of publications, international research hotspots, and direction of collaboration. The results of this study may provide information on knowledge graph, frontier trends and identify research topics in melanoma. More current research proved that ncRNA plays a crucial role in the biological behavior of melanoma including proliferation, invasion, metastasis, drug resistance, etc. With the development of research on ncRNA and melanoma, ncRNA may great potential in development of early diagnosis, targeted therapy and efficacy evaluation in the future. The results of this study also provide new perspectives and research partners for researchers in this field.

## Data Availability

The raw data supporting the conclusions of this article will be made available by the authors, without undue reservation.
